# Relationship between heifer carcass maturity and beef quality characteristics

**DOI:** 10.1093/tas/txaa017

**Published:** 2020-02-08

**Authors:** Kacie C Hoffman, Michael J Colle, James A Nasados, Sara J Gray, Jakobie Rogers, Jessie B Van Buren, Kendelle J Puga, Gordon K Murdoch, Ronald P Richard, Matthew E Doumit

**Affiliations:** Department of Animal and Veterinary Sciences, University of Idaho, Moscow, ID

**Keywords:** beef, carcass, heifer, maturity, palatability

## Abstract

Our objective was to determine the relationship between heifer carcass maturity and beef palatability of the longissimus lumborum (LM) and biceps femoris (BF). Left sides of A (*n* = 30), B (*n* = 30), and C (*n* = 30) maturity heifer carcasses under 30 mo of age by dentition were used. Carcasses were selected to ensure similar marbling scores across maturity groups (Small to Modest). Beef strip loins (LM) and outside rounds (BF) were obtained from these carcasses. Steaks were used to measure color stability, lipid oxidation (thiobarbituric acid reactive substances; TBARS), Warner-Bratzler shear force (WBSF), soluble and insoluble collagen, and consumer sensory perceptions. Heifer carcass maturity did not affect pH, fluid loss, WBSF, or collagen content of LM or BF steaks (*P* > 0.29). In LM and BF steaks, a maturity × day of retail display interaction occurred for TBARS, in which B maturity steaks had lower levels of lipid oxidation compared with A and C maturity steaks from the fourth day to the end of the retail display (*P* < 0.01). Nevertheless, LM steaks from B maturity carcasses tended to have lower overall acceptability (*P* = 0.08) and juiciness (*P* = 0.09) than steaks from C maturity carcasses, but steaks from B and C maturity carcasses did not differ from LM steaks obtained from A maturity carcasses. No differences in tenderness or flavor were observed due to maturity (*P* > 0.24). Similarly, maturity had no effect on sensory characteristics of BF steaks (*P* > 0.30). In conclusion, our results indicate that advanced physiological maturity does not decrease palatability of strip loin or outside round steaks from carcasses of heifers under 30 mo of age.

## INTRODUCTION

Recently, the USDA-AMS implemented new standards that allow for the use of age documentation, dentition, or physiological maturity to classify beef carcasses into maturity categories for quality grading (Federal Register, 2017). Before this change, USDA-AMS only allowed the use of physiological maturity as an indicator of carcass maturity when determining quality grade (USDA, 2016). When using physiological maturity, carcasses are designated to maturity groups A through E based on vertebral ossification, rib shape and size, and lean color and texture (USDA, 2016). Approximate ages associated with each of these maturity groups are A (9–30 mo), B (30–42 mo), C (42–72 mo), D (72–96 mo), and E (>96 mo) (Tatum, 2007).

The majority of conventionally raised heifer and steer carcasses fall within the A maturity category (Boykin et al., 2017). However, O’Connor et al. (2007) found that cattle between 22 and 24 mo of age have a 9% probability of producing B maturity carcasses and a 3% probability of producing a C maturity carcass. It is not uncommon for cattle under 30 mo of age, specifically heifers, to grade B maturity or older based on physiological maturity. It is well known that estrogen accelerates skeletal ossification, increasing the physiological maturity of heifer carcasses as compared with steers of a similar age (Shackelford et al., 1995; Field et al., 1997; Grumbach and Auchus, 1999). Tatum (2011) found that heifers under 30 mo of age are 7 times more likely to produce B maturity carcasses, and 11 times more likely to produce C maturity or older carcasses, as compared with steer counterparts. Additionally, Moore et al. (2012) reported that 46.7% of carcasses that graded Standard had Small marbling but were downgraded from low Choice based on designation as B maturity. In the summer of 2017, carcasses that quality graded Standard received an approximately $26.00 per hundredweight discount, while C maturity or older carcasses received a discount of nearly $39.00 per hundredweight, relative to Choice carcasses (USDA, 2017). The current research was conducted to determine if these discounts were warranted based on beef quality characteristics of carcasses from heifers verified to be under 30 mo of age by dentition.

## MATERIALS AND METHODS

Institutional Animal Care and Use Committee approval was not needed as no live cattle were used in this experiment. The University of Idaho Institutional Review Board certified this project as Exempt for human subject participation in consumer sensory analysis.

### Carcass Selection

The left sides of 90 beef heifer carcasses were selected on 4 d over a 3-mo period at a commercial beef processing facility (Toppenish, WA) in 2015 and 2016. Selected carcasses were from heifers finished in feedlots in the Western United States and Canada. All carcasses were determined to be from heifers less than 30 mo of age based upon dentition, though chronological age was unknown. Thirty carcasses within each physiological maturity category of A^00^–A^99^ (A), B^00^–B^99^ (B), and C^00^–C^99^ (C) were selected. Carcasses were also selected to ensure marbling scores of Small^00^ (SM) to Modest^99^ (MT). This resulted in carcasses of low or average Choice, Standard, Commercial Quality Grades (USDA, 2016).

Initial selection of carcasses was determined based on overall maturity evaluations from USDA graders, as well as marbling scores collected by an USDA-approved instrument grading system (e+v Technology GmbH & Co. KG, Oranienburg, Germany). Upon selection, carcasses were moved to stationary rails and carcass data (skeletal and lean maturity, marbling score, Quality Grade, hot carcass weight, ribeye area, 12th rib fat thickness, and Yield Grade) were collected by trained University of Idaho personnel utilizing USDA marbling cards, USDA ossification cards, measuring probe, and ribeye area grids. KPH was removed at the plant prior to selection of the carcasses and so could not be measured. An average of 2.5% KPH was used to determine the final Yield Grade for each carcass. Vacuum packaged outside rounds (Institutional Meat Purchase Specification [IMPS] 171B) and strip loins (IMPS 180; LM [longissimus lumborum]) from the left side of each carcass were purchased from AB Foods (Toppenish, WA) and transported chilled to the University of Idaho Meat Science Laboratory (Moscow, ID).

### Product Preparation

**Figure 1.**
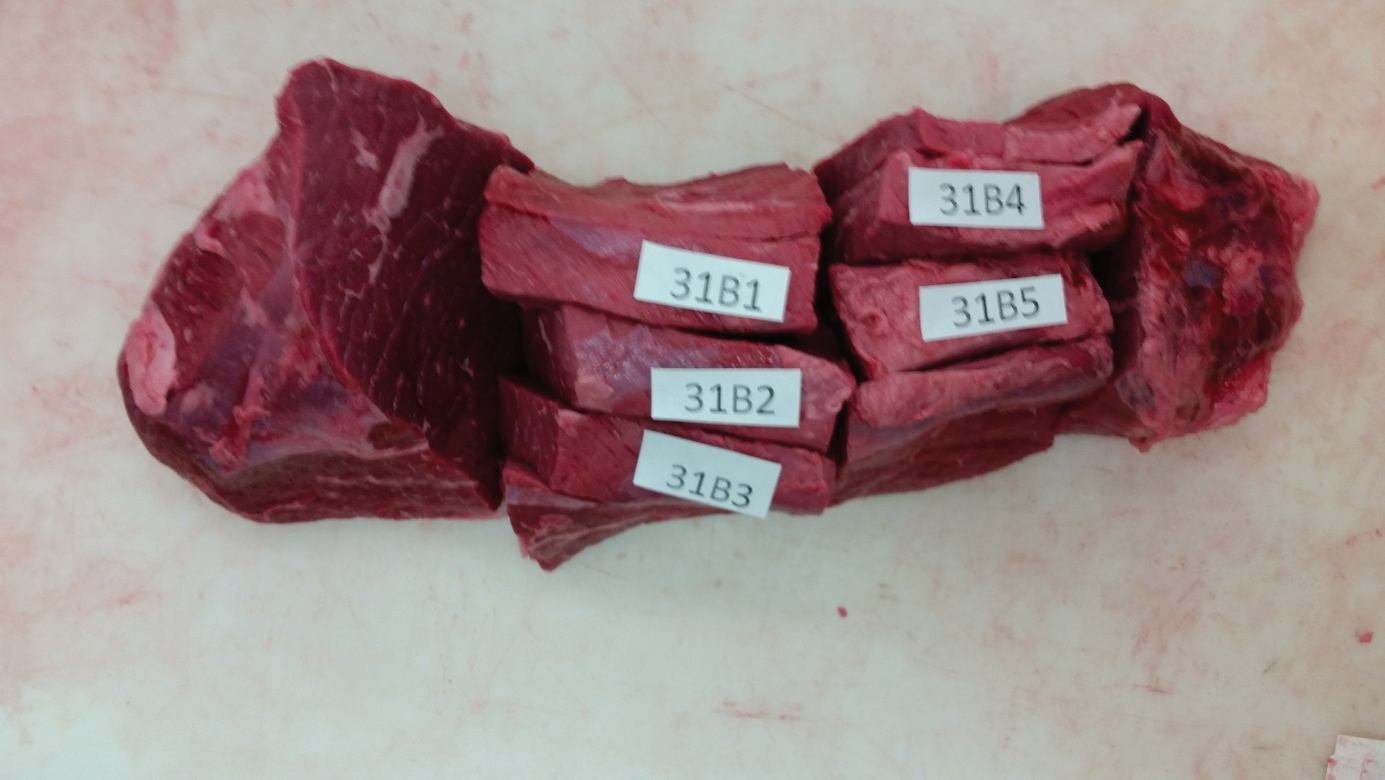
Outside round roast with the side muscle removed (IMPS 171D). Steaks were cut perpendicular to the to the grain of the muscle (IMPS 1171D) and are also referred to as a ‘“western griller steak”.

Subprimal cuts were stored in boxes under refrigerated conditions (2°C) and aged until 14 d postmortem. Subprimals were then removed from vacuum packaging and the ischiatic heads were removed from the outside rounds to produce trimmed outside round flats (IMPS 171D; BF) as described in the Institutional Meat Purchase Specifications (USDA, 2014). Five 2.54 cm steaks were then cut from the LM (IMPS 1180) and BF (IMPS 1171D; Fig. 1). Steaks were assigned by position to analyze retail shelf-life, lipid oxidation, Warner-Bratzler shear force (WBSF), insoluble and soluble collagen, and consumer acceptability.

### Retail Shelf-life

Steaks used for retail display were weighed, placed in white Styrofoam trays with the freshly cut surface exposed, and overwrapped with oxygen permeable PVC film (Koch Industries, Inc. #7500-3815; Wichita, KS). Once steaks had bloomed for at least 60 min, two objective color measurements per steak were taken using a Hunter MiniScan EZ (Restin, VA). These measurements represented day 0 of retail display. Subsequent measurements were taken on days 1 through 6. The Hunter MiniScan was equipped with a 25 mm-diameter measuring area and a 10° standard observer. The MiniScan was set to illuminant A and Commission International de l’Eclairage (CIE) L*, a*, and b* values were recorded. Each day prior to use the machine was calibrated against black and white calibration tiles. Subjective color measurements were taken daily for the 7-d retail display period by three evaluators following Section 7 Appendix C of the American Meat Science Association guidelines (AMSA, 2012). Oxygenated lean color (1 = extremely bright cherry-red, 2 = bright cherry-red, 3 = moderately bright cherry-red, 4 = slightly bright cherry-red, 5 = slightly dark cherry-red, 6 = moderately dark red, 7 = dark red, 8 = extremely dark red), amount of browning (1 = no evidence of browning, 2 = dull, 3 = grayish, 4 = brownish-gray, 5 = brown, 6 = dark brown), discoloration (1 = none, 2 = slight, 3 = small, 4 = moderate, 5 = extreme), surface discoloration (percentage metmyoglobin formation; 1 = no discoloration [0%], 2 = slight discoloration [1–20%], 3 = small discoloration [21–40%], 4 = modest discoloration [41–60%], 5 = moderate discoloration [61–80%], 6 = extensive discoloration [81–100%]), and color uniformity (1 = uniform, 2 = slight two-toning, 3 = small amount of two-toning, 4 = moderate two-toning, 5 = extreme two-toning) were measured, and averages of the three evaluators were utilized for analysis. Evaluators were familiarized with scoring and calibrated on day 0 of every retail display by reviewing images of LM and BF steaks.

Steaks were displayed in a glass retail case (Model GDM-69, True Manufacturing Co., O’Fallon, MO) kept at approximately 2 °C. The retail display case utilized natural white Hg 40w lights and the average light intensity was 409 lx. Steaks were rotated following daily measurements to minimize lighting and temperature effects due to location. Prior to the retail display steaks were weighed and following the 7-d display they were re-weighed to determine retail fluid loss.

### Lipid Oxidation

Thiobarbituric acid reactive substances (TBARS) were measured to determine the extent of lipid oxidation. The end (~1 cm) of the steak was discarded before samples were taken from the top half (exposed surface) of the steak avoiding the edges. Samples were cut by hand to approximately 0.5 cm wide, 2.0 cm long, and 1.27 cm thick. This initial sample represented day 0 of retail display. On days 2, 4, and 6 of retail display, TBARS were again taken to measure lipid oxidation. TBARS analysis followed the protocol defined in the Meat Color Measurement Guidelines (AMSA, 2012). During the 7-d period, steaks were displayed as listed above.

### Cooking

Steaks utilized for Warner-Bratzler Shear Force (WBSF) and collagen analysis were cooked following aging for 14 d postmortem. Steaks were weighed prior to and following cooking to measure fluid lost during the cooking process. Steaks were cooked on open-hearth broilers to an internal temperature of 40 °C, then were flipped and cooked to a final internal temperature of 71 °C. Temperatures of steaks were measured using hypodermic temperature probes (Omega Engineering Co.) coupled with a 12-channel scanning thermocouple thermometer (Digi-Sense, Cole-Parmer Instrument Co.). Probes were inserted into the geometric center of the steak.

### Warner-Bratzler Shear Force

Prior to coring for WBSF, steaks were cooled at 4 °C overnight. Six cores (1.27-cm diameter) were mechanically removed per steak, parallel with muscle fiber orientation, using a drill-mounted coring device (GR Manufacturing, Manhattan, KS). Shear force was then determined by shearing cores once through the center, perpendicular to muscle fiber orientation, using a Warner-Bratzler shearing machine (GR Manufacturing, Manhattan, KS). The six shear values were averaged to determine a shear force (kg) for each steak.

### Collagen Solubility

Remaining portions of WBSF steaks were diced, placed in 50 ml centrifuge tubes and frozen at −20 °C until collagen analysis could be completed. Eight randomly selected LM and BF steaks from each maturity category (A, B, and C) were used to determine soluble and insoluble collagen, as described by Colle et al. (2015). Total collagen was determined by adding soluble and insoluble collagen values.

### pH

A portable pH meter (Model SevenGo, Mettler Toledo, Woburn, MA) equipped with an InLab Solids Pro puncture-type electrode was used to measure pH. The pH meter was calibrated each day prior to use with standard pH 4.0 and pH 7.0 buffers. LM and BF steak pH was measured prior to steaks being cooked for consumer sensory analysis. The pH was taken at the edge of the steak. This edge was removed prior to cubing for sensory analysis to avoid potential effects of probe insertion on palatability.

### Consumer Sensory Perceptions

Two sensory panels were conducted: one for strip loin and one for outside round steaks. Steaks used for sensory panels were individually vacuum packaged after aging and frozen at −20 °C until needed. Steaks were thawed at 4 °C for 24 h prior to the panel and then cooked as previously described. Cooking order of steaks within A, B, and C maturities was randomized. Four (1.27 cm × 1.27 cm × steak thickness) cubes were obtained per steak and placed in covered cups assigned with a random number. Cups were held in insulated containers with hot packs until ready to serve. Consumer panelists were asked to evaluate samples for overall acceptability, tenderness, juiciness, and flavor using a 9-point scale (9 = like extremely, extremely tender, extremely juicy, and like flavor extremely, respectively; 1 = dislike extremely, not at all tender, extremely dry, dislike flavor extremely, respectively). Panelists (*n* = 72 per muscle) were presented with and evaluated five samples in a random order from A, B, and C maturities using an incomplete block design. Additionally, for each sample panelists were asked if they would be willing to purchase the product, if an off-flavor was present, and what the most and least liked attribute was (tenderness, juiciness, flavor, or texture/mouth feel), if applicable. Sensory panels were held at the Washington State University Sensory Evaluation Facility (Pullman, WA) which utilizes divided sensory booths and white lighting. Both panels began at 10:00 a.m. and ended upon reaching the desired number of panelists.

### Statistical Analysis

Data were analyzed using the Mixed Model procedure of the Statistical Analysis System (SAS Institute, Inc., Cary, NC). Subprimal cuts (LM and BF) served as experimental units and maturity and day of retail display, as well as their interaction, served as fixed variables. All color and TBARS measurements were analyzed as repeated measures. Consumer sensory panel analysis was set up using an incomplete block design. Differences in least square means were compared using the DIFF option. Significance was determined at *P* ≤ 0.05, and data were considered trending at *P* ≤ 0.10.

## RESULTS AND DISCUSSION

### Characteristics of Selected Carcasses

Carcass measurements used to determine Quality Grade and Yield Grade of heifer carcasses (*n* = 90) can be found in Table 1. By design, skeletal maturity differed (*P* < 0.01) between A, B, and C maturity heifer carcasses. Skeletal maturity of carcasses ranged from A^20^ to C^90^ with a mean of B^55^. However, lean maturity measurements were considerably more youthful than skeletal maturity, with a range of A^20^ to B^90^ and a mean of A^78^. These results are similar to those of Boykin et al. (2017) who determined, based on National Beef Quality Audit data from 2016, that the mean lean maturity score of U.S. fed beef (*n* = 8,741) was A^55^, while skeletal maturity was A^69^. Likewise, Semler et al. (2016) determined that the overall mean skeletal and lean maturity for beef carcasses (*n* = 600) from fed cattle under and over 30 mo of age was B^54^ and A^50^, respectively. As animals increase in chronological age, myoglobin content in muscle increases, leading to a darker red oxygenated lean color (Breidenstein et al., 1968; USDA, 2016). In the current research, C maturity carcasses had lower lean maturity values than B maturity carcasses (*P* < 0.05). While this result was unexpected, chronological age likely had little effect on lean color among carcasses from these heifers under 30 mo of age.

**Table 1.** USDA Quality Grade and Yield Grade carcass measurements*

	Maturity				
	A	B	C		
Measurement	*n* = 30	*n* = 30	*n* = 30	SEM	*P*
Skeletal maturity^†^	167^c^	249^b^	348^a^	4	<0.01
Lean maturity^†^	177^ab^	188^a^	170^b^	5	0.05
Overall maturity^†^	174^c^	220^b^	300^a^	3	<0.01
Marbling score^‡^	494	502	501	6	0.57
Hot carcass weight, kg	402	419	413	7	0.20
12th rib fat thickness, cm	1.85	1.70	1.83	0.13	0.67
Ribeye area, cm^2^	37.34	37.34	39.12	0.76	0.24
Yield grade^||^	3.5	3.5	3.4	0.2	0.83

^a–c^Means in the same row within a measurement that do not share a common superscript letter differ.

*Data presented collected by trained personnel.

^†^Maturity: A maturity = 100–199, B = 200–299, C = 300–399.

^‡^Marbling score: Small = 400–499, Modest = 500–599.

^||^An average of 2.5% kidney, pelvic, and heart fat (KPH) was used to determine Yield Grade.

When determining overall maturity, the US Grading Standards for Carcass Beef (USDA, 2016) state that when differences occur between skeletal and lean maturity, more emphasis is placed on skeletal maturity. The standards also indicate that overall maturity must not differ from the skeletal maturity by more than one full grade. In the current research this accounts for numerically lower degrees of overall maturity for B and C maturity carcasses compared with A maturity carcasses. As expected, A, B, and C maturity carcasses differed (*P* < 0.01) in overall maturity (Table 1). Mean overall maturities for A, B, and C maturity carcasses were A^74^, B^20^, and C^00^, respectively.

Marbling scores from the 90 heifer carcasses ranged from SM^30^ to MT^70^ with a mean of SM^99^. By design, carcass marbling scores were similar (*P* = 0.57) across A, B, and C maturity groups (Table 1). The USDA Quality Grade distributions of the 90 carcasses collected were 26.7% Average Choice, 26.7% Low Choice, 13.3% Standard, and 33.3% Commercial (Table 2). Had these carcasses been graded using the modernized beef grading standards, 52.2% would have been categorized as Average Choice based on their marbling scores (Table 2).

**Table 2.** Quality Grade distribution by maturity*

	Maturity			
*n* = 90	A	B	C	All
*Utilizing physiological maturity*				
Average Choice	43.3	36.7	—	26.7
Low Choice	56.7	23.3	—	26.7
Standard	—	40.0	—	13.3
Commercial	—	—	100.0	33.3
*Utilizing modernized Standards for Carcass Beef*				
Average Choice	43.3	60.0	53.3	52.2
Low Choice	56.7	40.0	46.7	47.8

*Carcasses selected to ensure Small to Modest marbling.

Carcass measurements used to determine Yield Grade (hot carcass weight, adjusted 12th rib fat thickness, and ribeye area) did not differ (*P* ≥ 0.20) between A, B, and C maturity carcasses (Table 1). As animals increase in age, an increase in carcass weight, ribeye area, and lipid deposition are usually observed (Duarte et al., 2011). However, since cattle in the current study were all verified to be under 30 mo of age by dentition, and therefore are of similar age, we would expect for A, B, and C maturity carcasses to have comparable mean hot carcass weights, ribeye areas, and adjusted 12th rib fat measurements.

### Longissimus Lumborum and Biceps Femoris Quality Characteristics

Increases in physiological maturity have been associated with decreases in beef tenderness (Berry et al., 1974; Miller et al., 1983; Weston et al., 2002). This decrease in tenderness is associated with more mature collagen crosslinks and more heat stable collagen (Tatum, 2011). Shorthose and Harris (1990) determined that age-associated toughening of beef was more pronounced in collagen rich muscles than muscles having lower collagen concentrations. In the current research, LM and BF subprimals were obtained to represent product that contain relatively low and high levels of connective tissue, respectively. Mean total collagen content of LM and BF steaks was 7.9 ± 2.8 and 10.5 ± 3.1 mg collagen/g meat, respectively. However, no differences (*P* ≥ 0.47) in insoluble or total collagen were found between A, B, and C maturity carcasses from LM or BF steaks (Table 3).

**Table 3.** pH, purge, Warner-Bratzler shear force, cook loss, insoluble, and total collagen of longissimus lumborum and biceps femoris steaks

		Maturity				
Attribute	*n*	A	B	C	SEM	*P*
Longissimus lumborum						
pH	90	5.49	5.51	5.50	0.01	0.60
Percent purge	90	1.37	1.26	1.33	0.06	0.46
WBSF, kg	90	3.82	4.05	3.89	0.14	0.49
Percent cook loss	90	23.37	24.97	22.79	1.08	0.34
Insoluble collagen, mg/g	24	6.33	7.73	7.44	0.85	0.48
Total collagen, mg/g	24	6.94	8.53	8.32	1.40	0.47
Biceps femoris						
pH	90	5.51	5.51	5.52	0.01	0.65
Percent purge	90	1.74	1.81	1.65	0.10	0.48
WBSF, kg	90	3.70	3.87	3.55	0.14	0.29
Percent cook loss	90	28.99	29.44	28.62	0.72	0.72
Insoluble collagen, mg/g	24	9.54	9.55	9.80	1.14	0.98
Total collagen, mg/g	24	10.60	10.41	10.60	1.25	0.99

Furthermore, no differences (*P* ≥ 0.29) in WBSF values of LM or BF steaks from A, B, or C maturity carcasses were found (Table 3). Steaks with WBSF values below 4.6 kg are typically considered tender by consumers (Shackelford, 1991). In the current research, mean overall WBSF values for LM and BF steaks were 3.9 and 3.7 kg, respectively. Although mean WBSF measurements of BF steaks were numerically lower than LM steaks, Rhee et al. (2004) demonstrated that the BF ranked last among 11 muscles in trained sensory panel tenderness, but also ranked fourth in tenderness based on WBSF. Our data are consistent with Rhee et al. (2004) who concluded that WBSF is more useful to study changes in tenderness within muscles than among different muscles. Similarly, sensory panel tenderness scores in the current experiment were lower for BF than LM.

The pH of meat can play a large role in fluid loss, meat color, and tenderness. Muscle pH is approximately 7.0 and after death normally declines to 5.4–5.5 (Wulf et al., 2002). If ultimate pH remains above 6.0, a product defect referred to as a dark cutter or dark, firm, and dry (DFD) may result. These products are noted for their dark color, high water holding capacity, and also a decrease in product tenderness (Lawrie, 1958; Wulf et al., 2002; Bass et al., 2010; English et al., 2016). Because of these factors, dark cutting carcasses were avoided for this experiment. No differences were found (*P* ≥ 0.60) in pH of LM or BF steaks from A, B, or C maturity carcasses (Table 3). Mean pH values for LM and BF steaks were 5.50 and 5.51, respectively, with the highest steak pH recorded at 5.71. Since meat pH did not differ among maturity groups, difference reported for lean color are likely due to factors other than pH.

Over the 6-d retail display, interactions were detected between day of retail display and maturity for objective color measurements of LM and BF steaks (Tables 4 and 5). Redness (a*) and lightness (L*) of many steaks increased from day 0 to 1 of retail display, even though steaks were allowed to bloom for at least 60 min prior to taking the day 0 objective color measurement. McKenna et al. (2005) also reported increasing a* values from day 0 to day 1 of retail display. They attributed the increase in redness to high levels of oxygen consumption that occur early in display and do not allow for the steak to fully bloom. On day 0 of retail display, B maturity LM steaks had higher (*P* < 0.01) a* values than C maturity LM steaks (Table 4). Similarly, on the final day of retail display, B maturity LM steaks had a redder lean color than both A and C maturity LM steaks. These results confirm carcass lean maturity values, where B maturity carcasses had a darker red lean color as compared with C maturity carcasses. However, BF steaks from B maturity carcasses had lower (*P* < 0.01) a* values (less red lean color) than C maturity BF steaks on the second day of display and had lower a* values than A maturity BF steaks on the last day of retail display (Table 5). B maturity LM steaks had higher L* values (*P* < 0.01) than A maturity LM steaks on day 0 of retail display. On day 2 of retail display, C maturity LM steaks had significantly higher b* values versus A maturity steaks. A similar change in b* values occurred in BF steaks, where C maturity steaks were more yellow in color than A and B maturity steaks.

**Table 4.** Instrument color of longissimus lumborum steaks

		Maturity			
*n* = 90	Day of display	A	B	C	SEM
Longissimus lumborum					
^†^L*	0	37.07^bwy^	38.69^ay^	37.71^abwy^	0.57
*P* < 0.01	1	39.73^z^	40.16^z^	40.55^zx^	0.57
	2	36.55^y^	35.70^x^	37.23^yz^	0.57
	3	38.09^x^	38.20^y^	38.49^yx^	0.57
	4	37.94^x^	38.22^y^	38.26^yz^	0.57
	5	37.57^wx^	37.97^y^	38.55^xy^	0.57
	6	37.49^wx^	38.36^y^	37.64^wz^	0.57
^†^a*	0	34.72^abz^	36.09^az^	34.33^by^	0.59
*P* < 0.01	1	34.88^z^	35.19^z^	35.50^z^	0.59
	2	31.41^y^	31.26^y^	32.77^x^	0.59
	3	30.73^xy^	29.74^x^	30.49^w^	0.59
	4	31.09^xy^	30.90^y^	31.51^wx^	0.59
	5	29.97^x^	30.37^xy^	30.27^w^	0.59
	6	25.45^bw^	27.64^aw^	25.59^bv^	0.59
^†^b*	0	29.89^z^	30.88^z^	29.95^z^	0.51
*P* = 0.03	1	28.12^y^	28.65^y^	29.06^z^	0.51
	2	25.93^bx^	26.37^abx^	27.53^ay^	0.51
	3	24.92^w^	24.02^w^	24.60^w^	0.51
	4	25.03^wx^	24.68^w^	26.00^x^	0.51
	5	24.36^w^	24.82^w^	25.13^wx^	0.51
	6	22.67^v^	22.80^v^	23.43^v^	0.51

^a–b^Within a row, means without a common letter differ.

^v–z^Within a column, muscle, and trait, means without a common letter differ.

^†^L* (lightness), a* (redness), and b* (yellowness).

**Table 5.** Instrument color of biceps femoris steaks

		Maturity			
*n* = 90	Day of display	A	B	C	SEM
Biceps femoris					
^†^L*	0	37.43	38.75	37.47	0.60
*P* = 0.27	1	38.89	38.99	39.58	0.60
	2	35.52	34.72	35.45	0.60
	3	36.11	35.97	35.85	0.60
	4	35.58	36.33	35.35	0.60
	5	35.48	35.71	35.05	0.60
	6	35.30	36.53	36.66	0.60
^†^a*	0	34.97^z^	36.19^z^	35.2^z^	0.67
*P* < 0.01	1	32.93^y^	32.59^y^	33.8^y^	0.67
	2	27.77^abx^	26.68^bx^	29.37^ax^	0.67
	3	24.48^w^	23.36^w^	24.48^w^	0.67
	4	23.85^w^	23.89^w^	24.12^w^	0.67
	5	20.80^u^	21.48^v^	21.25^v^	0.67
	6	22.59^av^	20.62^bv^	22.05^abv^	0.67
^†^b*	0	31.04^z^	31.88^z^	31.39^z^	0.49
*P* = 0.01	1	28.00^y^	27.81^y^	28.98^y^	0.49
	2	25.49^bx^	24.77^bx^	26.89^ax^	0.49
	3	22.5^vw^	21.53^v^	22.34^v^	0.49
	4	23.01^v^	22.77^w^	23.49^w^	0.49
	5	21.74^w^	21.53^uv^	22.23^v^	0.49
	6	21.62^w^	20.57^u^	21.56^v^	0.49

^a–b^Within a row, means without a common letter differ.

^u–z^Within a column, muscle, and trait, means without a common letter differ.

^†^L* (lightness), a* (redness), and b* (yellowness).

Subjective color measurements (oxygenated lean color, amount of browning, discoloration, surface discoloration, and color uniformity) did not differ among A, B, and C maturity LM steaks (*P* ≥ 0.37; data not included). However, a maturity by day of retail display interaction was observed for oxygenated lean color of BF steaks (*P* = 0.04; Table 6). On the third day of retail display, B maturity BF steaks had higher (worse) oxygenated lean color scores than C maturity steaks, while A maturity steaks did not differ from either. Similarly, on the fourth day of retail display B maturity BF steaks had higher oxygenated lean color scores than A and C maturities (5.82 vs. 5.32 and 5.24, respectively).

**Table 6.** Visual color of biceps femoris steaks

		Maturity			
*n* = 90	Day of display	A	B	C	SEM
Biceps femoris					
Oxygenated lean color*	0	3.1^v^	3.1^u^	2.8^u^	0.2
*P* = 0.04	1	3.5^w^	3.6^v^	3.2^v^	0.2
	2	4.3^x^	3.2^w^	4.2^w^	0.2
	3	5.1^aby^	5.4^ax^	4.7^bx^	0.2
	4	5.3^by^	5.8^ay^	5.2^by^	0.2
	5	5.8^z^	6.1^z^	5.8^z^	0.2
	6	6.0^z^	6.3^z^	6.0^z^	0.2

^a–b^Within a row, means without a common letter differ.

^u–z^Within a column and trait, means without a common letter differ.

*1 = extremely bright cherry red, 2 = bright cherry red, 3 = moderately bright cherry red, 4 = slightly bright cherry red, 5 = slightly dark cherry red, 6 = moderately dark red, 7 = dark red, 8 = extremely dark red.

Oxygen on the cut surface of steaks can lead to autoxidation of lipids through the presence of free-radicals. Other factors that can lead to lipid oxidation are heat and light, catalysts, fatty acids present, and pH. A commonly used assay to quantify lipid oxidation is thiobarbituric acid reactive substances (TBARS) analysis. This measures malondialdehyde (MDA) which is an end product of lipid oxidation. A maturity by day of retail display interaction was observed (*P* < 0.01) for TBARS values (lower mg MDA/kg of meat) of LM and BF steaks. Less lipid oxidation occurred at days 4 and 6 of retail display in B maturity LM steaks than A and C maturity LM steaks (Tables 2 and 7). Throughout the 6-d retail display period, lipid oxidation of LM steaks from B maturity carcasses did not increase, whereas A maturity LM steaks showed increased oxidation on the last day of retail display, and C maturity LM steaks lipid oxidation increased significantly by day 4 of retail display. Similarly, B maturity BF steaks had significantly less lipid oxidation by days 4 and 6 of retail display than BF steaks from A and C maturity carcasses. Mean lipid oxidation of all steaks was below 1.0, except for A maturity BF steaks on the final day of retail display. This is noteworthy since this level of lipid oxidation has been associated with off-flavors (McKenna et al., 2005). However, in normal retail display settings, steaks overwrapped in oxygen permeable film are usually removed from the shelf within 72 h due to oxidation of myoglobin that results in a brown color considered unacceptable by consumers (Delmore, 2009). By 72 h of retail display, LM and BF steak lipid oxidation levels were well below 1.0.

**Table 7.** Lipid oxidation of longissimus lumborum and biceps femoris steaks*

		Maturity			
*n* = 90	Day of display	A	B	C	SEM
Longissimus lumborum	0	0.24^y^	0.21	0.20^x^	0.03
*P* < 0.01	2	0.22^y^	0.19	0.23^x^	0.03
	4	0.25^ay^	0.22^b^	0.27^ay^	0.03
	6	0.33^az^	0.23^b^	0.32^az^	0.03
Biceps femoris	0	0.43^w^	0.42^x^	0.40^w^	0.06
*P* < 0.01	2	0.63^x^	0.50^x^	0.62^x^	0.06
	4	0.80^ay^	0.62^by^	0.79^ay^	0.06
	6	1.01^az^	0.74^bz^	0.94^az^	0.06

^a–b^Within a row, means without a common letter differ.

^w–z^Within a column, muscle, and trait, means without a common letter differ.

*mg malondialdehyde/kg meat.

During the LM sensory panel, no differences were detected (*P* ≥ 0.25) in tenderness or flavor between steaks from A, B, or C maturity carcasses (Table 8). However, overall acceptability (*P* = 0.08) and juiciness (*P* = 0.09) tended to be higher in LM steaks from C maturity carcasses than B maturity, but steaks from B and C maturity carcasses did not differ from those of A maturity carcasses. Similarly, Acheson et al. (2014) and Semler et al. (2016) found no differences (*P* > 0.05) in LM tenderness, juiciness, or flavor between steaks from A and B or older maturity carcasses. Additionally, in the current research LM steaks from C maturity carcasses received the highest percentage of consumer panelists indicating a willingness to purchase the product (Table 9). Consumers did not report differences (*P* ≥ 0.31) in acceptability, tenderness, juiciness, or flavor for BF steaks from A, B, or C maturity carcasses (Table 8).

**Table 8.** Consumer panel analysis of longissimus lumborum and biceps femoris steaks (*n* = 72 panelists per muscle)*

	Maturity				
*n* = 90	A	B	C	SEM	*P*
Longissimus lumborum					
Acceptability	5.7	5.4	5.9	0.1	0.08
Tenderness	5.7	5.4	5.8	0.2	0.29
Juiciness	5.2	5.1	5.6	0.2	0.09
Flavor	5.5	5.5	5.8	0.2	0.25
Biceps femoris					
Acceptability	5.2	4.9	4.9	0.2	0.31
Tenderness	4.7	4.4	4.5	0.2	0.51
Juiciness	4.6	4.4	4.7	0.2	0.39
Flavor	5.3	5.2	5.2	0.2	0.86

*Scale, 9 = like extremely, extremely tender, extremely juicy, and like flavor extremely, respectively; 1 = dislike extremely, not at all tender, extremely dry, and dislike flavor extremely, respectively.

**Table 9.** Consumer panel preferences for longissimus lumborum and biceps femoris steaks (*n* = 72 panelists per muscle)

	Longissimus lumborum			Biceps femoris		
	Maturity			Maturity		
Consumer preferences	A	B	C	A	B	C
Like most*						
Flavor	33.3	44.9	41.4	58.7	45.6	44.8
Tenderness	34.2	24.3	27.9	23.1	12.6	16.7
Juiciness	21.6	19.6	23.4	12.5	24.3	22.9
Texture	10.8	11.2	7.2	5.8	17.5	15.6
Like least^†^						
Flavor	33.6	24.8	30.8	16.9	20.7	20.7
Tenderness	25.2	27.4	25.2	44.1	40.5	46.8
Juiciness	27.1	30.1	29.9	21.2	24.0	25.2
Texture	14.0	17.7	14.0	17.8	14.9	7.2
Off flavor^‡^						
Yes	24.4	24.2	21.2	22.7	25.2	23.3
No	75.6	75.8	78.8	77.3	74.8	76.7
Purchase^||^						
Yes	57.6	54.6	68.1	51.7	45.8	42.4
No	42.4	45.4	31.9	48.3	54.2	57.6

*Percentage of panelists that liked that attribute the most.

^†^Percentage of panelists that liked that attribute the least.

^‡^Percentage of panelists that did or did not detect an off flavor.

^||^Percentage of panelists willing to or not willing to purchase the product.

### Importance to Industry

The devaluation of carcasses that are under 30 mo of age by dentition, but downgraded due to advanced physiological maturity, was typically in excess of $20 per hundredweight prior to the modernization of the carcass beef standards. Recently, Acheson et al. (2014) used A and B–C maturity steer and heifer carcasses from cattle under 30 mo of age to determine if differences occurred in sensory properties of the LM. The authors concluded that carcasses with similar marbling scores from grain-finished cattle younger than 30 months of age by dentition have similar palatability regardless of maturity. Semler et al. (2016) furthered this research by using steer and heifer carcasses to determine if dentition of carcasses 30 mo or older and carcasses under 30 mo better segregated carcasses based on sensory properties. Similarly, these authors found few differences in palatability of carcasses under 30 mo of age regardless of physiological maturity.

The current research specifically utilized heifer carcasses, since they tend to show advanced physiological maturity at younger chronological ages compared with steers. However, other factors, such as pregnancy, spaying, usage of anabolic implants, as well as severity of anabolic implants can also influence degree of skeletal maturation (Tatum, 2011). Little background information is known on the 90 selected heifer carcasses, with the exception that heifers were not spayed, but did receive anabolic implants and were grain-finished. Very few differences were determined between LM and BF steaks from A, B, and C maturity carcasses under 30 mo of age. Based on the results of this study, discounts were not warranted for heifer carcasses under 30 mo of age by dentition with small or modest marbling. These results support prior research (Acheson et al., 2014; Semler et al., 2016), in that carcasses from cattle harvested at less than 30 mo of age based on dentition produce beef that provides the same shelf-life and eating experience regardless of its physiological maturity. Furthermore, these results align with the recent modernization of the U.S. standards for grades of carcass beef, which states all carcasses under 30 mo of age by dentition should be considered A maturity.

## References

[CIT0001] Acheson R. J., Woerner D. R., and Tatum J. D. 2014 Effects of USDA carcass maturity on sensory attributes of beef produced by grain-finished steers and heifers classified as less than 30 months old using dentition. J. Anim. Sci. 92:1792–1799. doi:10.2527/jas.2013-7553. 24663159

[CIT0002] AMSA 2012 Meat color measurement guidelines. Champaign (IL): American Meat Science Association.

[CIT0003] Bass P. D., Engle T. E., Belk K. E., Chapman P. L., Archibeque S. L., Smith G. C., and Tatum J. D. 2010 Effects of sex and short-term magnesium supplementation on stress responses and longissimus muscle quality characteristics of crossbred cattle. J. Anim. Sci. 88:349–360. doi:10.2527/jas.2009-2264. 19783692

[CIT0004] Berry B. W., Smith G. C., and Carpenter Z. L. 1974 Beef carcass maturity indicators and palatability attributes. J. Anim. Sci. 38:507–514. doi:10.2527/jas1974.383507x.

[CIT0005] Boykin C. A., Eastwood L. C., Harris M. K., Hale D. S., Kerth C. R., Griffin D. B., Arnold A. N., Hasty J. D., Belk K. E., Woerner D. R., et al. 2017 National beef quality audit-2016: in-plant survey of carcass characteristics related to quality, quantity, and value of fed steers and heifers. J. Anim. Sci. 95:2993–3002. doi:10.2527/jas.2017.1543. 28727109

[CIT0006] Breidenstein B. B., Cooper C. C., Cassens R. G., Evans G., and Bray R. W. 1968 Influence of marbling and maturity on the palatability of beef muscle. I. Chemical and organoleptic considerations. J. Anim. Sci. 27:1532–1541. doi:10.25257/jas1968.2761532x.

[CIT0007] Colle M. J., Richard R. P., Killinger K. M., Bohlscheid J. C., Gray A. R., Loucks W. I., Day R. N., Cochran A. S., Nasados J. A., and Doumit M. E. 2015 Influence of extended aging on beef quality characteristics and sensory perception of steaks from the gluteus medius and longissimus lumborum. Meat Sci. 110:32–39. doi:10.1016/j.meatsci.2015.06.013. 26172241

[CIT0008] Delmore R. J 2009 Beef Shelf-life. White Paper. Cattlemen’s Beef Board and National Cattlemen’s Beef Association [accessed April 20, 2015]. Available from https://www.beefresearch.org/CMDocs/BeefResearch/Beef%20Shelf-life.pdf.

[CIT0009] Duarte M. S., Paulino P. V., Fonseca M. A., Diniz L. L., Cavali J., Serão N. V., Gomide L. A., Reis S. F., and Cox R. B. 2011 Influence of dental carcass maturity on carcass traits and meat quality of Nellore bulls. Meat Sci. 88:441–446. doi:10.1016/j.meatsci.2011.01.024. 21333459

[CIT0010] English A. R., Wills K. M., Harsh B. N., Mafi G. G., VanOverbeke D. L., and Ramanathan R. 2016 Effects of aging on the fundamental color chemistry of dark-cutting beef. J. Anim. Sci. 94:4040–4048. doi:10.2527/jas.2016-0561. 27898916

[CIT0011] Federal Register 2017 Notice: United States standards for grades of carcass beef. Livest. Seed Program. Agric. Market. Serv [accessed September 25, 2016]. Available from https://www.federalregister.gov/documents/2017/06/19/2017–12647/united-states-standards-for-grades-of-carcass-beef.

[CIT0012] Field R., McCormick R., Balasubramanian V., Sanson D., Wise J., Hixon D., Riley M., and Russell W. 1997 Tenderness variation among loin steaks from A and C maturity carcasses of heifers similar in chronological age. J. Anim. Sci. 75:693–699. doi:10.2527/1997.753693x. 9078485

[CIT0014] Grumbach M. M., and Auchus R. J. 1999 Estrogen: consequences and implications of human mutations in synthesis and action. J. Clin. Endocrinol. Metab. 84:4677–4694. doi:10.1210/jcem.84.12.6290. 10599737

[CIT0015] Lawrie R. A 1958 Physiological stress in relation to dark-cutting beef. J. Sci. Food Agric. 9:721–727. doi:10.1002/jsfa.2740091106.

[CIT0016] McKenna D. R., Mies P. D., Baird B. E., Pfeiffer K. D., Ellebracht J. W., and Savell J. W. 2005 Biochemical and physical factors affecting discoloration characteristics of 19 bovine muscles. Meat Sci. 70:665–682. doi:10.1016/j.meatsci.2005.02.016. 22063894

[CIT0017] Miller R. K., Tatum J. D., Cross H. R., Bowling R. A., and Clayton R. P. 1983 Effects of carcass maturity on collagen solubility and palatability of beef from grain-finished steers. J. Food Sci. 48:484–486, 525. doi:10.1111/j.1365-2621.1983.tb10772.x.

[CIT0018] Moore M. C., Gray G. D., Hale D. S., Kerth C. R., Griffin D. B., Savell J. W., Raines C. R., Belk K. E., Woerner D. R., Tatum J. D., et al. 2012 National beef quality audit-2011: in-plant survey of targeted carcass characteristics related to quality, quantity, value, and marketing of fed steers and heifers. J. Anim. Sci. 90:5143–5151. doi:10.2527/jas.2012-5550. 22952369

[CIT0019] O’Connor M. E., Ransom J. R., and Feil M. 2007 USDA physiological maturity validation study: validating the relationship between chronological age and physiological maturity in the U.S. fed-beef population. [accessed April 23, 2015]. Available from http://www.maff.go.jp/j/syouan/douei/beef_taiou/pdf/080131b.pdf.

[CIT0020] Rhee M. S., Wheeler T. L., Shackelford S. D., and Koohmaraie M. 2004 Variation in palatability and biochemical traits within and among eleven beef muscles. J. Anim. Sci. 82:534–550. doi:10.2527/2004.822534x. 14974553

[CIT0021] Semler M. L., Woerner D. R., Belk K. E., Enns K. J., and Tatum J. D. 2016 Effects of United States Department of Agriculture carcass maturity on sensory attributes of steaks produced by cattle representing two dental age classes. J. Anim. Sci. 94:2207–2217. doi:10.2527/jas.2016-0382. 27285716

[CIT0022] Shackelford S. D., Koohmaraie M., and Wheeler T. L. 1995 Effects of slaughter age on meat tenderness and USDA carcass maturity scores of beef females. J. Anim. Sci. 73:3304–3309. doi:10.2527/1995.73113304x. 8586588

[CIT0023] Shackelford S. D., Morgan J. B., Cross H. R., and Savell J. W. 1991 Identification of threshold levels for Warner-Bratzler shear force in beef top loin steaks. J. Muscle Foods. 2:289–296. doi:10.1111/j.1745-4573.1991.tb00461.x.

[CIT0024] Shorthose W. R., and Harris P. V. 1990 Effect of animal age on the tenderness of selected beef muscles. J. Food Sci. 55:1–8. doi:10.1111/j.1365-2621.1990.tb06004.x

[CIT0025] Tatum J. D 2007 Beef grading. Cattlemen’s Beef Board and National Cattlemen’s Beef Association [accessed April 20, 2015]. Available from https://www.beefresearch.org/CMDocs/BeefResearch/Beef%20Grading.pdf.

[CIT0026] Tatum J. D 2011 Animal age, physiological maturity, and associated effects on beef tenderness. Cattlemen’s Beef Board and National Cattlemen’s Beef Association. [accessed April 20, 2015]. Available from http://www.beefresearch.org/CMDocs/BeefResearch/PE_White_%20Papers/Animal_Age.pdf.

[CIT0027] USDA 2014 Institutional meat purchase specifications: fresh beef-series 100. Livest. Seed Program. Agric. Market. Serv. [accessed April 20, 2015]. Available from https://www.ams.usda.gov/sites/default/files/media/IMPS_100_Fresh_Beef%5B1%5D.pdf.

[CIT0028] USDA 2016 United States standards for grades of carcass beef. Livest. Seed Program. Agric. Market Serv [accessed January 5, 2018]. Available from https://www.ams.usda.gov/sites/default/files/media/CarcassBeefStandard.pdf.

[CIT0029] USDA 2017 National weekly direct slaughter cattle – premiums and discounts. Market News Serv [accessed June 12, 2017]. Available from https://mpr.datamart.ams.usda.gov/htmlResults.do?pk=39165702&path=Products\Cattle\Weekly%20Cattle\(LM_CT155)%20National%20Weekly%20Direct%20Slaughter%20Cattle%20-%20Premiums%20and%20Discounts.

[CIT0030] Weston A. R., Rogers R. W., and Althen T. G. 2002 Review: the role of collagen in meat tenderness. Professional Anim. Sci. 18:107–111. doi:10.15232/S1080-7446(15)31497-2.

[CIT0031] Wulf D. M., Emnett R. S., Leheska J. M., and Moeller S. J. 2002 Relationships among glycolytic potential, dark cutting (dark, firm, and dry) beef, and cooked beef palatability. J. Anim. Sci. 80:1895–1903. doi:10.2527/2002.8071895x. 12162657

